# Supplementary figures and images for: Analysis of ancestry heterozygosity suggests that hybrid incompatibilities in threespine stickleback are environment dependent
Source: PLoS Biol. 2022 Jan 10;20(1):e3001469. doi: 10.1371/journal.pbio.3001469 (PMC8746713; doi:10.1371/journal.pbio.3001469)

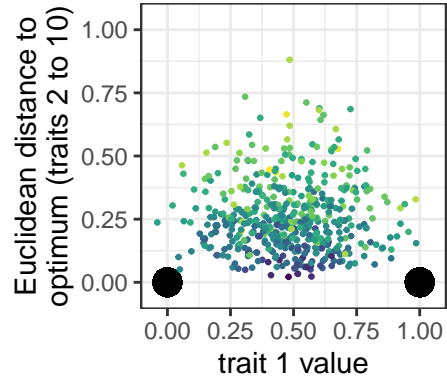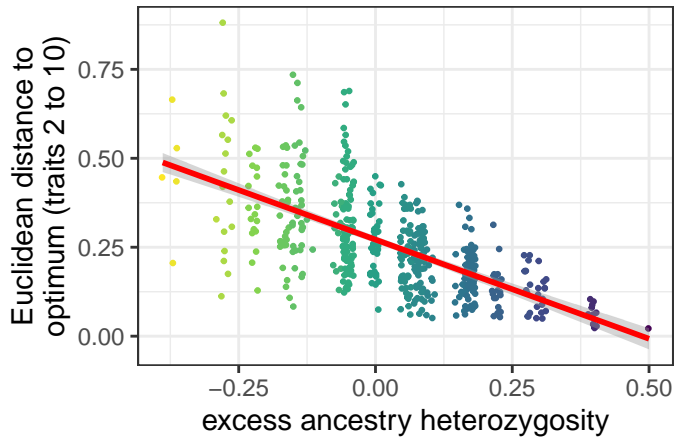

Supplement: S1 Fig — The model is as in Fig 1 in the main text except there are 10 traits instead of 2 and the optimum of the adapting population is “0” for traits 2 to 9. Plots and model are inspired by Fig 1 in [18]. Both panels depict results from a representative simulation run of adaptive divergence and hybridization between 2 populations. Colored points are individual hybrids, with darker colors indicating higher heterozygosity. The left panel depicts the distribution of 500 F2 hybrid phenotypes where the x-axis depicts the value of the selected trait, and the y-axis depicts the Euclidean distance from the optimum for traits 2–9 (i.e., y = ∑i = 210‍zi2). Large black points are the 2 parent phenotypes. The right panel depicts the relationship between excess ancestry heterozygosity and the maladaptive distance from the optimum for individual hybrids [13]. Points are slightly jittered horizontally. The plot shows that this maladaptive trait expression is lower in F2s with greater excess ancestry heterozygosity. Heterozygosity values are fairly discrete because a small number of loci underlie adaptation in the plotted simulation run. The data and code required to recreate this figure may be found at https://doi.org/10.5061/dryad.h18931zn3. (PDF) [file pbio.3001469.s001.pdf]

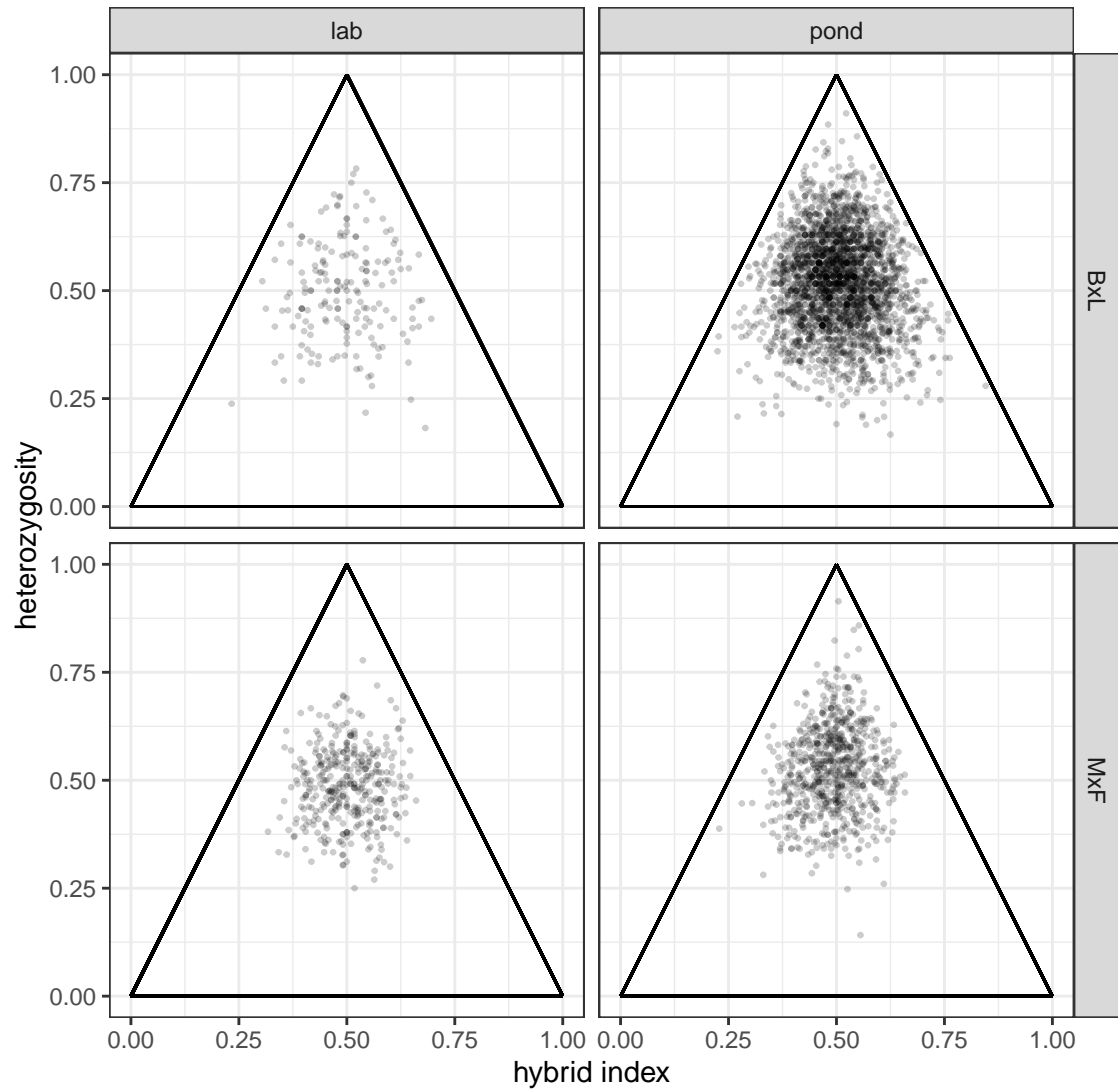

Supplement: S2 Fig — Each point represents an individual hybrid and shows each individual’s hybrid index (frequency of benthic or marine alleles in its genome) and its mean heterozygosity. Hybrid index and heterozygosity are used because many loci are being considered simultaneously. These graphs are not used for analysis, but rather are shown to allow readers to visualize the structure of the raw data that underlies our analysis. Specifically, the shapes of the distributions of heterozygosity and hybrid index values are similar between environments and crosses—the means are just subtly different. The data and code required to recreate this figure may be found at https://doi.org/10.5061/dryad.h18931zn3. (PDF) [file pbio.3001469.s002.pdf]

mean excess  
ancestry heterozygosity  $\pm$  1 SE

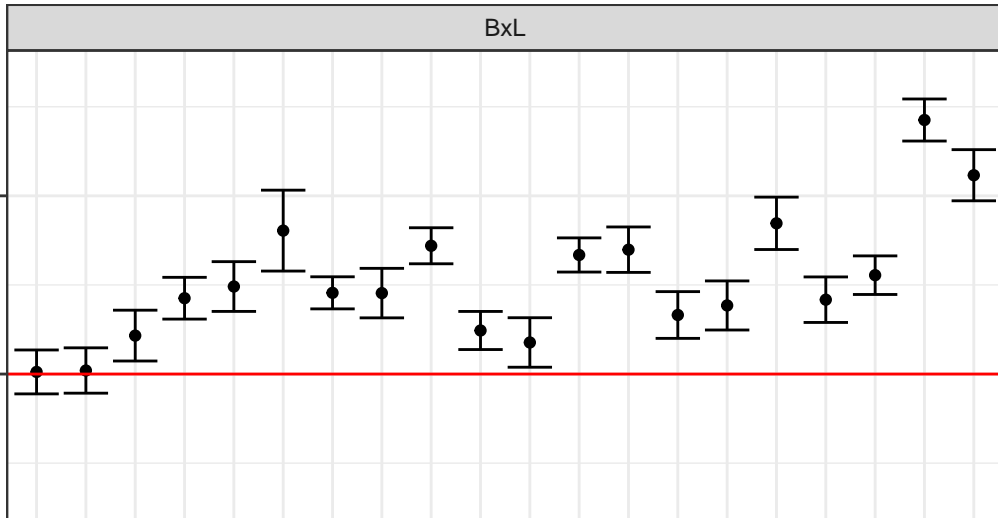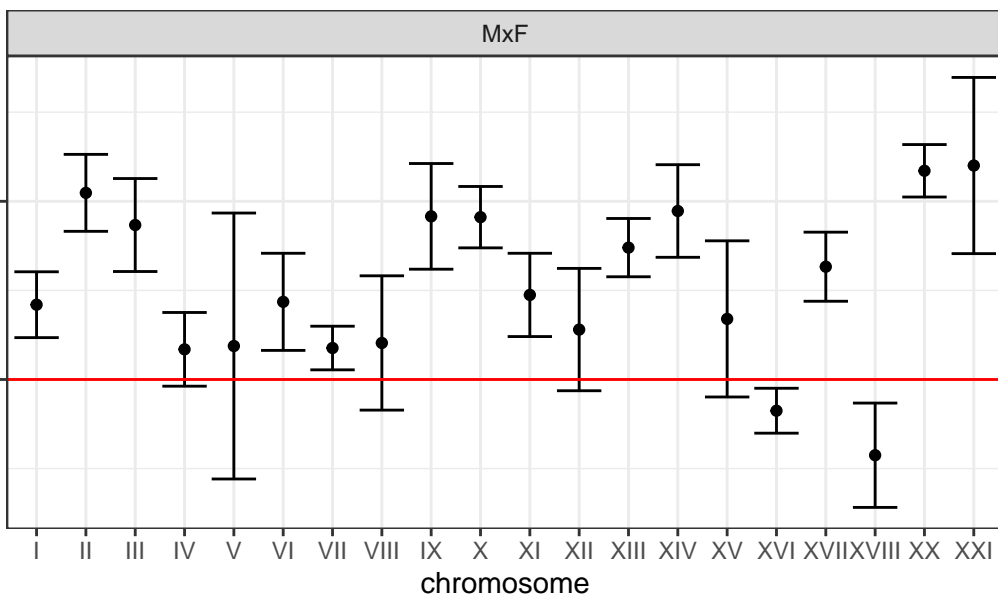

Supplement: S3 Fig — Each point is the average excess ancestry heterozygosity for all loci on a given chromosome (raw values; not residuals from a statistical model). Linkage group XIX contains the sex determining region and is plotted or analyzed. Error bars are 1 SE. The data and code required to recreate this figure may be found at https://doi.org/10.5061/dryad.h18931zn3. (PDF) [file pbio.3001469.s003.pdf]

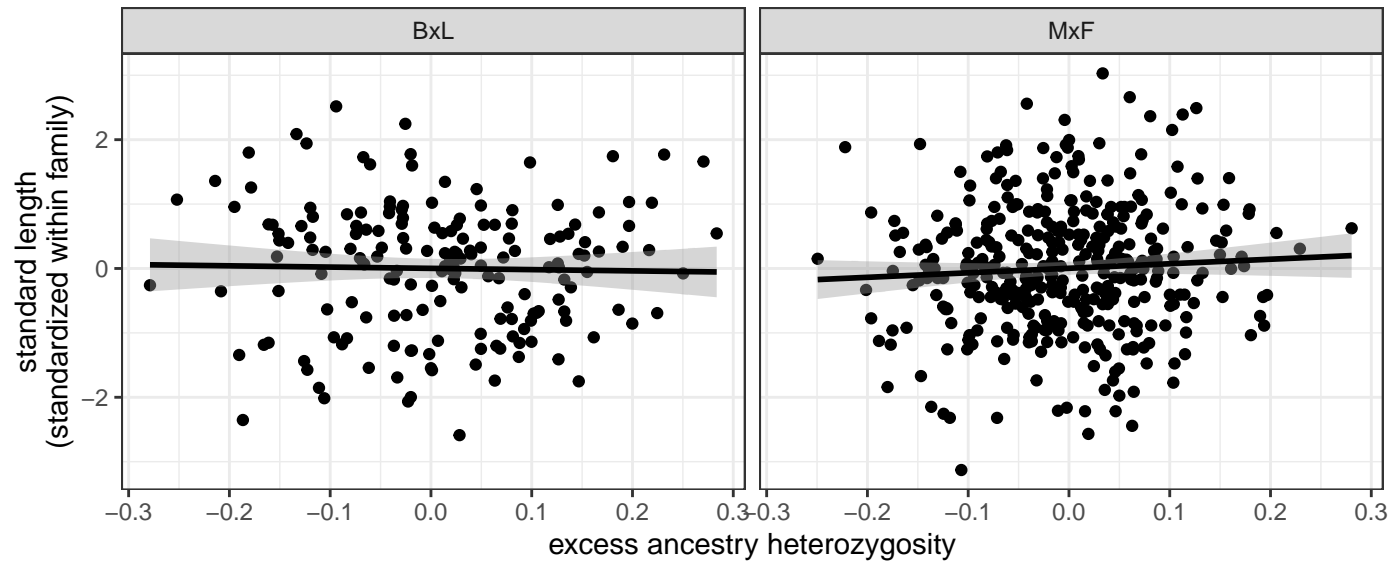

Supplement: S4 Fig — Results are residuals from visreg [31]. Each point is an individual F2 hybrid. Standard length is standardized within family (1 family each for Paxton and Priest lakes for B×L and 4 families for M×F. The interaction between lake-of-origin mean heterozygosity was nonsignificant so we plot the main effect across both lakes of origin (Paxton and Priest). Mean heterozygosity was not significantly associated with standard length for either cross (B×L—β^ = −0.05 ± 0.67 [SE], F1,174 = 0.0075, P = 0.93; M×F—β^ = 0.65 ± 0.59 [SE], F1,372 = 1.24, P = 0.26). Analyses considering body depth (either individually or in a combined metric of “overall size”) give the same qualitative result (see archived R script). The data and code required to recreate this figure may be found at https://doi.org/10.5061/dryad.h18931zn3. (PDF) [file pbio.3001469.s004.pdf]

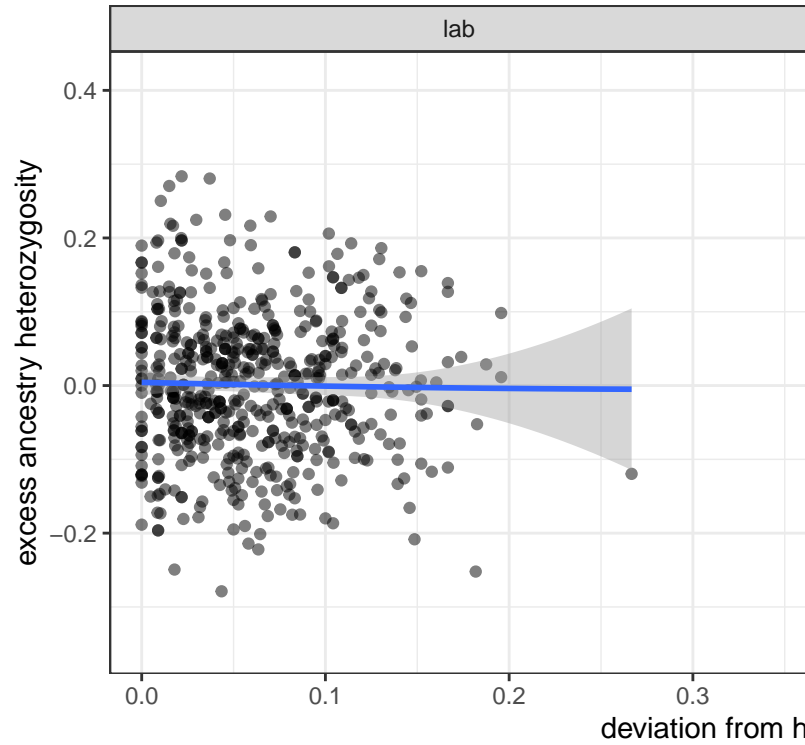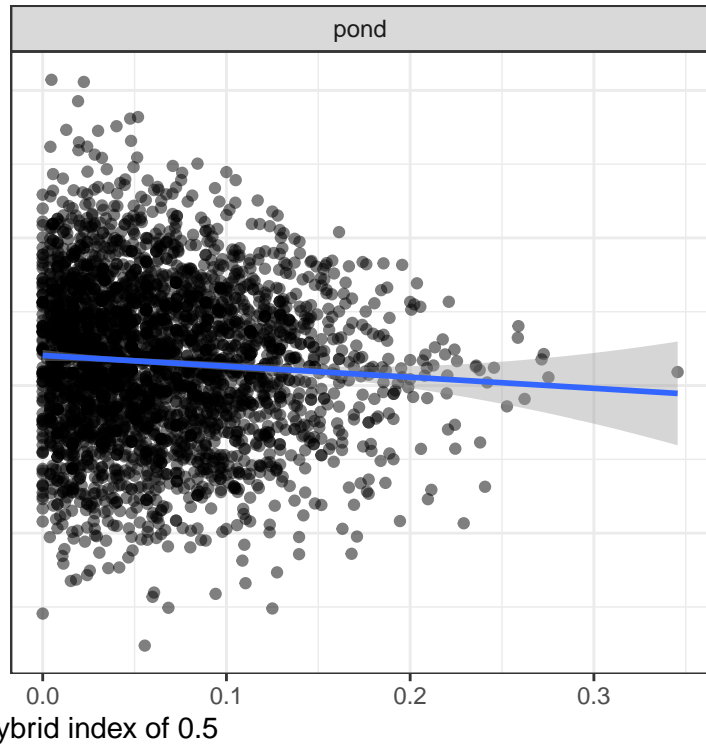

Supplement: S5 Fig — Each point is an individual recombinant hybrid and data are pooled across cross types. Excess ancestry heterozygosity declines as the hybrid index of pond-raised individuals deviates from 0.5 (Spearman ρ = −0.059; P = 0.0006), whereas there is no relationship in the lab (ρ = −0.012; P = 0.77). Bootstrap tests indicate that these 2 correlations are statistically indistinguishable, so we consider this analysis to be interesting and consistent with our hypothesis, but not conclusive. The data and code required to recreate this figure may be found at https://doi.org/10.5061/dryad.h18931zn3. (PDF) [file pbio.3001469.s005.pdf]

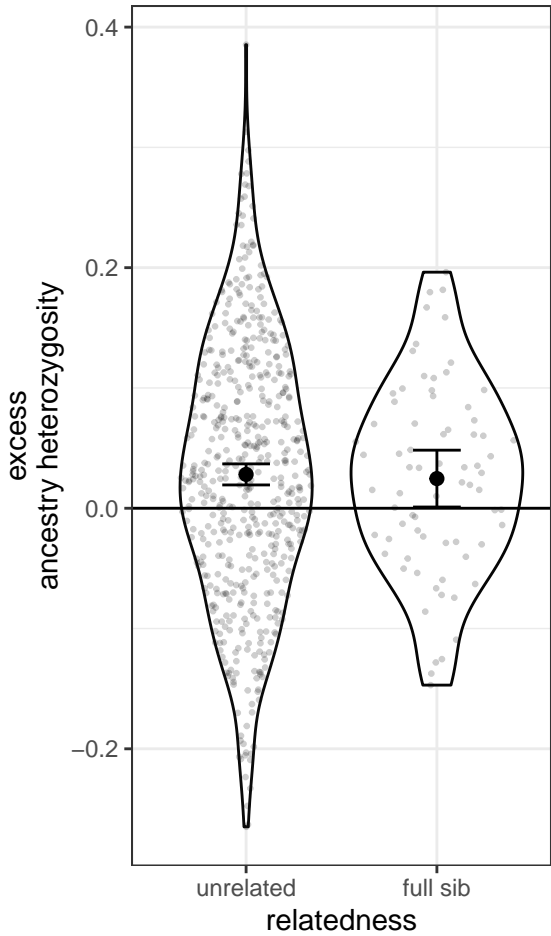

Supplement: S6 Fig — There is no difference in mean excess ancestry heterozygosity between F2 hybrids whose parents were unrelated and those whose parents were full siblings. Data from [11]. The data and code required to recreate this figure may be found at https://doi.org/10.5061/dryad.h18931zn. (PDF) [file pbio.3001469.s006.pdf]

excess ancestry heterozygosity

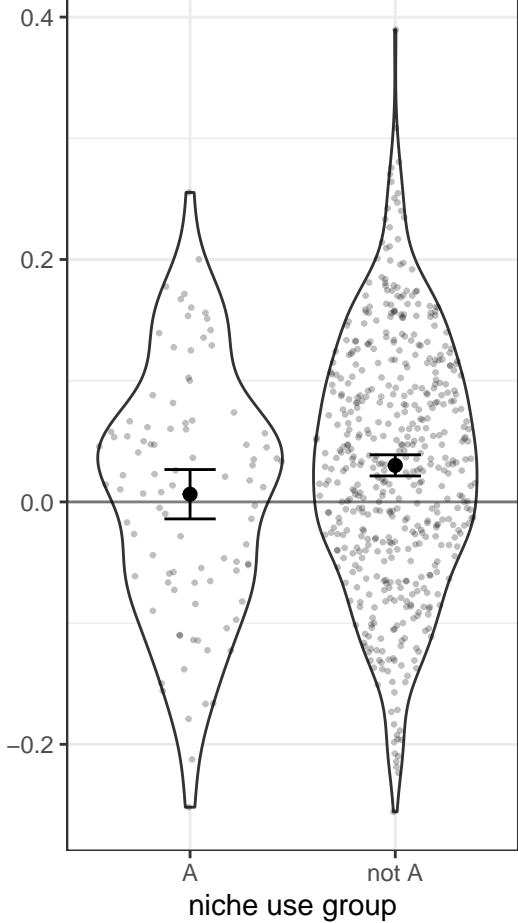

Supplement: S7 Fig — Assignments are from [11] and methods are described therein. Each point is an individual F2 hybrid. This result implies that phenotypically mismatched individuals have lower excess ancestry heterozygosity than nonmismatched individuals. The data and code required to recreate this figure may be found at https://doi.org/10.5061/dryad.h18931zn3. (PDF) [file pbio.3001469.s007.pdf]

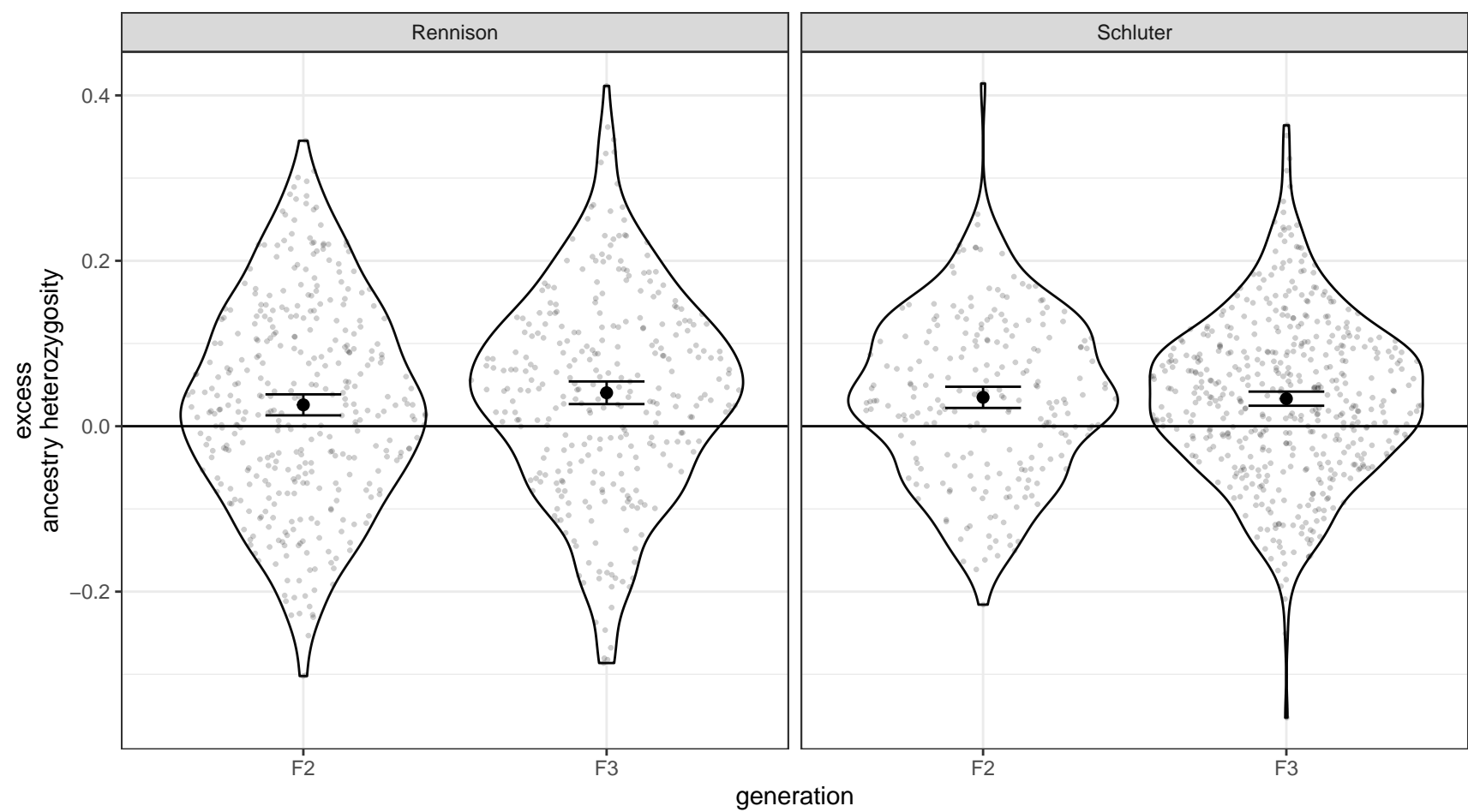

Supplement: S8 Fig — The plots show individual excess ancestry heterozygosity from the 2 studies that genotyped both the F2 and F3 generations [28,30]. The means (black dots, ± 95% CI) do not differ between generations in either study (Rennison group difference = 0.014 ±0.0095 [SE], F1,667 = 2.35, P = 0.13; Schluter group difference = 0.0016 ±0.0078 [SE], F1,721 = 0.042, P = 0.84). The data and code required to recreate this figure may be found at https://doi.org/10.5061/dryad.h18931zn3. (PDF) [file pbio.3001469.s008.pdf]

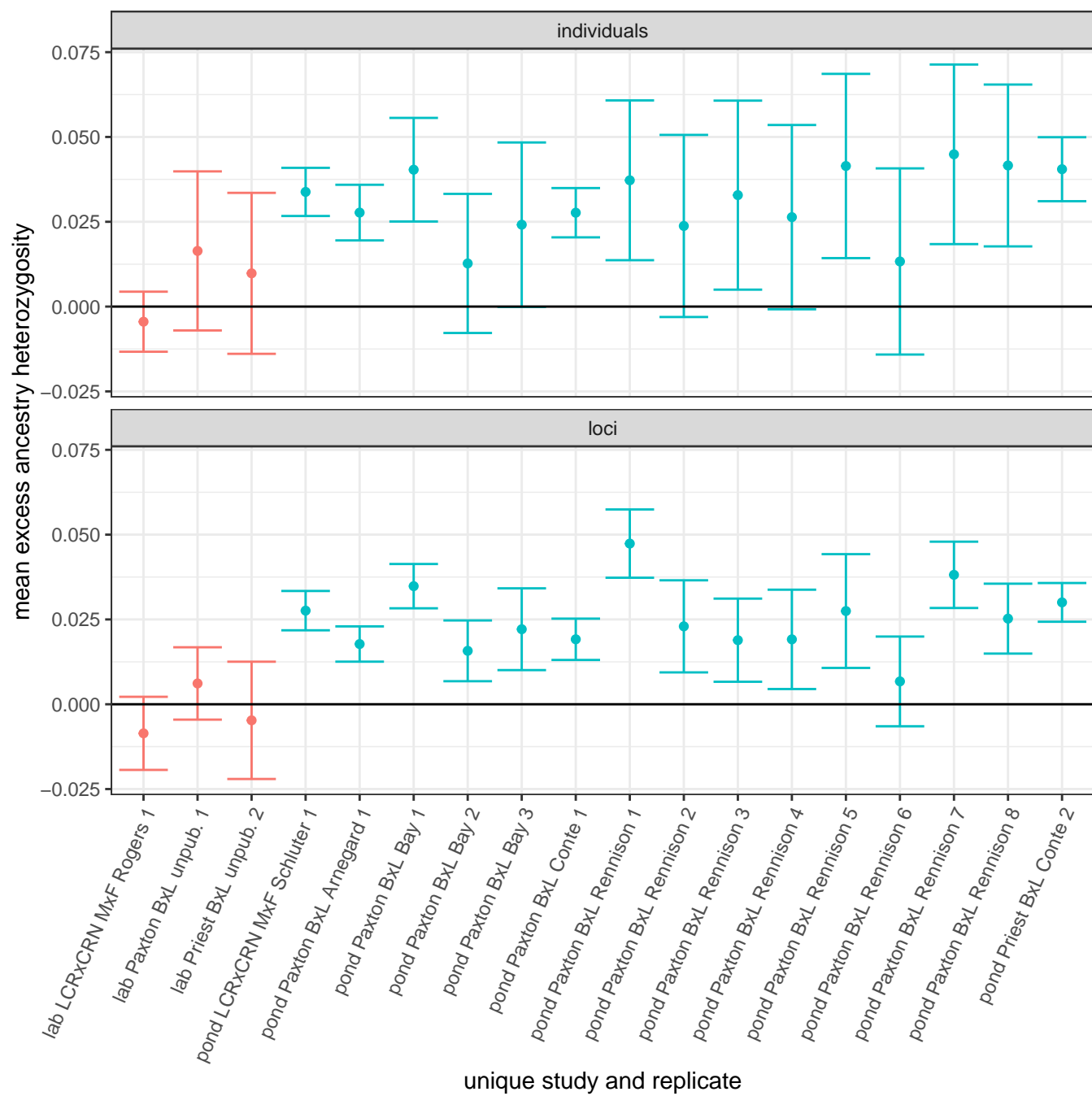

Supplement: S9 Fig — We consider a replicate to be a unique biparental F0 cross for aquarium studies and a unique pond for pond studies. Mean excess ancestry heterozygosity is shown for each such replicate for both individuals (upper) and loci (lower). In each panel, the horizontal line indicates no excess ancestry heterozygosity. Red points are “lab” replicates, and blue points are “pond” replicates. The data and code required to recreate this figure may be found at https://doi.org/10.5061/dryad.h18931zn3. (PDF) [file pbio.3001469.s009.pdf]

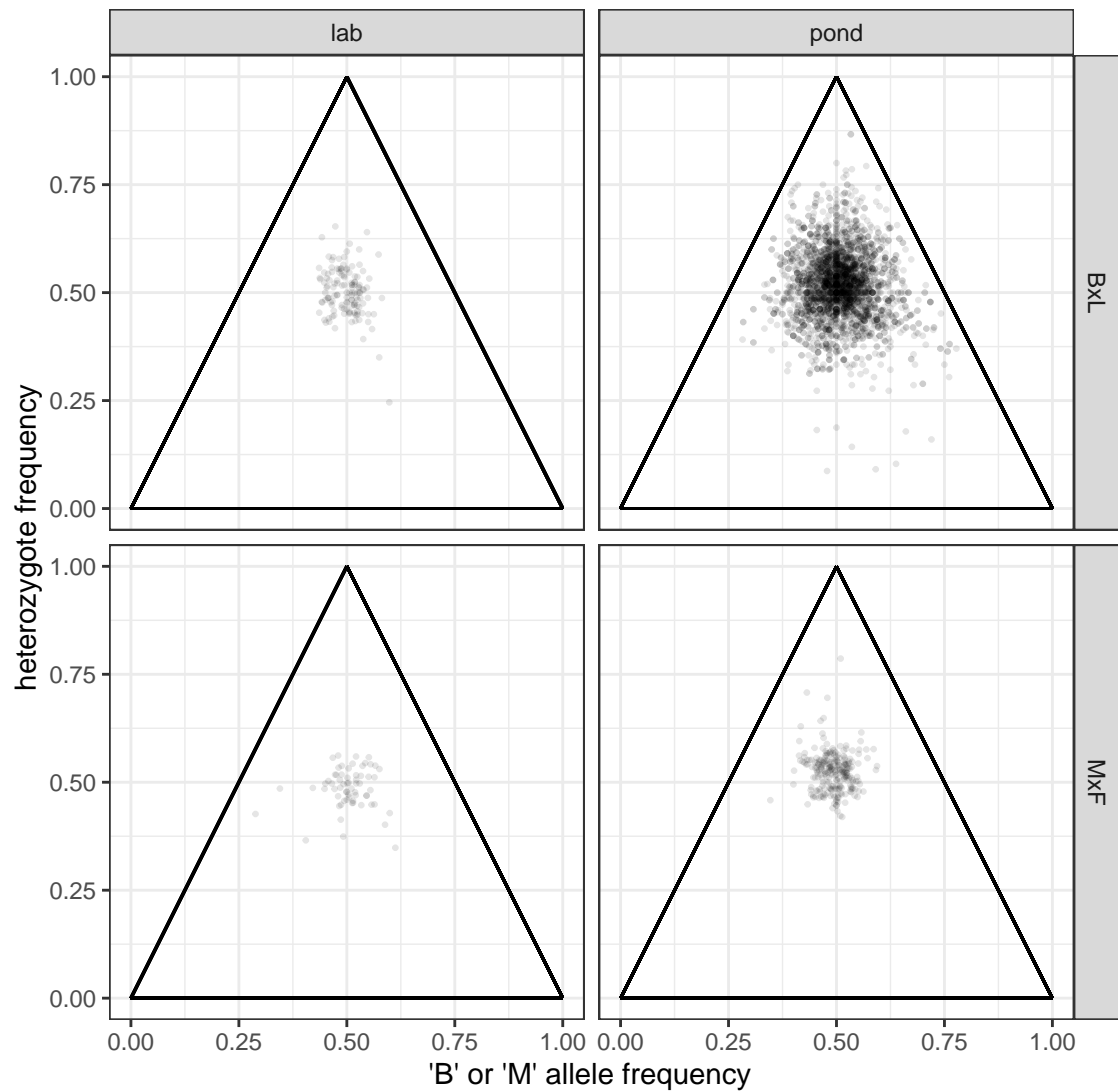

Supplement: S10 Fig — Each point represents genotyped locus within a given study (i.e., line in Table 1 in the main text) and shows the frequency of either benthic or marine alleles on the x-axis and its heterozygosity on the y-axis. These graphs are not used for analysis, but rather are shown to allow readers to visualize the structure of the raw data that underlies our analysis. Specifically, the shapes of the distributions of heterozygosity and hybrid index values are similar between environments and crosses—the means are just subtly different. The data and code required to recreate this figure may be found at https://doi.org/10.5061/dryad.h18931zn3. (PDF) [file pbio.3001469.s010.pdf]

**A**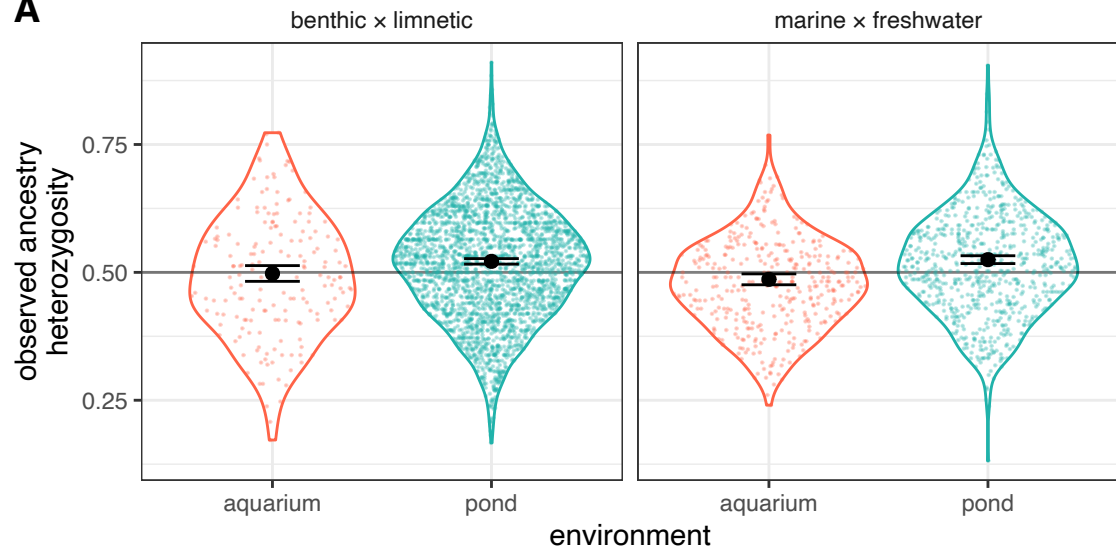**B**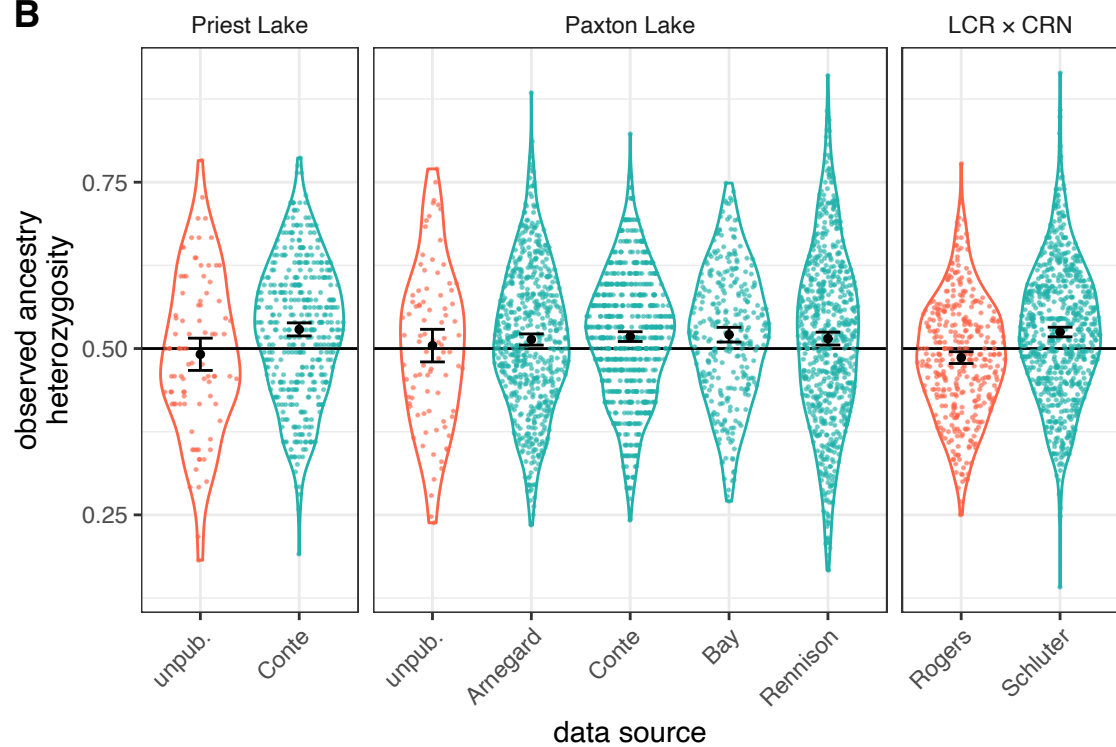

Supplement: S11 Fig — For full details, see caption of Fig 2 in the main text. Qualitative conclusions of statistical models are identical to those of the main analysis (see archived R script). The data and code required to recreate this figure may be found at https://doi.org/10.5061/dryad.h18931zn3. (PDF) [file pbio.3001469.s011.pdf]

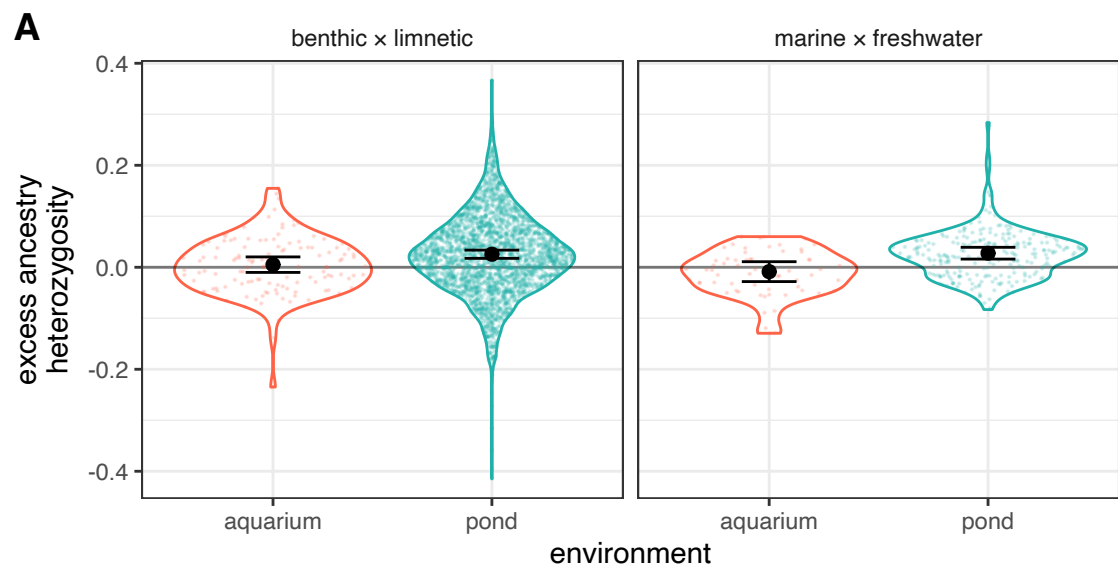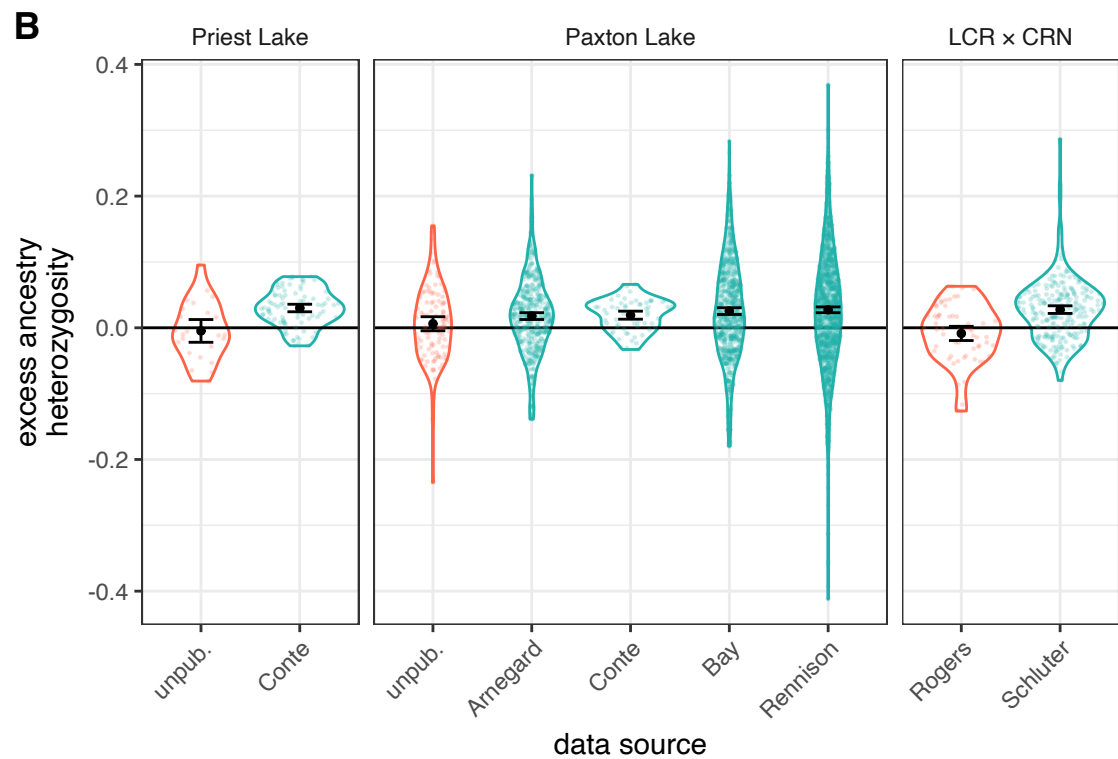

Supplement: S12 Fig — Figure is as in Fig 2 in the main text (and S11 Fig) and qualitative conclusions of statistical models are identical to those of the main analysis (see archived R script). The data and code required to recreate this figure may be found at https://doi.org/10.5061/dryad.h18931zn3. (PDF) [file pbio.3001469.s012.pdf]
