# Supplementary material for: Analysis of ancestry heterozygosity suggests that hybrid incompatibilities in threespine stickleback are environment dependent
Source: PLoS Biol. 2022 Jan 10;20(1):e3001469. doi: 10.1371/journal.pbio.3001469 (PMC8746713; doi:10.1371/journal.pbio.3001469)
Supplement: Attachment — Submitted filename: GEEI_R1_R2R_FINAL.pdf [file pbio.3001469.s013.pdf]

Response to the reviewers

Dear Dr. Roberts,

We are delighted that the reviewers found merit in our study and are glad to have the opportunity to revise our manuscript to be considered for publication in *PLOS Biology*. In line with the instructions, we have reproduced the entire text of the decision letter in this document and have made changes to the manuscript that are detailed in our responses to the relevant changes. The data underlying our manuscript is uploaded for reviewers, and if our manuscript is accepted we will archive all data and analysis code on Dryad.

On behalf of all authors,

Ken A. Thompson

Editor

Editor Point E 1.1 — Thank you very much for submitting your manuscript "Genetic evidence for environment-dependent hybrid incompatibilities in threespine stickleback" for consideration as a Short Reports at PLOS Biology. Your manuscript has been evaluated by the PLOS Biology editors, an Academic Editor with relevant expertise, and by three independent reviewers.

In light of the reviews (below), we are pleased to offer you the opportunity to address the comments from the reviewers in a revised version that we anticipate should not take you very long. We will then assess your revised manuscript and your response to the reviewers' comments and we may consult the reviewers again.

IMPORTANT: You'll see that the reviewers are all very positive about your study, but each has a number of requests for improvement. The Academic Editor supplied the following additional advice that you might find useful when prioritising your revisions: "I think reviewer #2 raises some interesting points about interpretation that need to be addressed. In particular his/her suggestion that inbreeding might lead to similar patterns seems valid to me—I think the authors need to address this in the discussion. I would be less concerned about some of the points raised by reviewer #3, such as using a different set of genetic markers, which would represent a significant additional burden of work. I think this goes beyond the scope of this paper, but the implications of possible biases in RAD-seq should be discussed."

Reply: We thank the editor, associate editor, and reviewers for evaluating our manuscript. In response to the specific comments, we have added analyses that address the Reviewer #2's point about inbreeding. We did not take all of the suggestions of Reviewer #3 but do note in the text that because the RAD-seq and SNP array are consistent with one another, this indicates that potential problems with RAD-based sequencing methods are unlikely to be responsible for our results.

Reviewer 1

Reviewer Point R 1.1 — I really appreciated this paper addressing the interesting question of coarse effects of incompatibilities across the genome. I found it concise and well written. I like the approach and the use of the existing data on sticklebacks to explore the interplay between incompatibilities and the environment. I only have a few minor questions/comments.

Reply: We are glad the reviewer found our analyses to be of value and enjoyed reading the manuscript.

Reviewer 1 Minor Points

Reviewer Point R 1.2 — L73: ”relatively homozygous” − > relatively more homozygous?

Reply: We have made the change exactly as suggested.

Reviewer Point R 1.3 — L98 ”interactions between individual hybrids”: do you mean competition and/or sexual selection? It might be worth adding some detail. Additionally, biotic interactions can also happen with parents don't you think? I do not grasp here what you want to convey by separating individual hybrids from other abiotic and biotic factors.

Reply: We meant to make a broad statement that hybrid incompatibilities can be "...caused by interactions between individuals and the complex ecological environments that they inhabit...". We meant for the text to indicate the situations hybrids find themselves in under natural contexts are responsible for selection on incompatibilities. In any case, this sentence has been removed through revision.

Reviewer Point R 1.4 — L339 about yeast: I am not a yeast expert at all, but I remember there can be frequent aneuploidies in some crosses. Is it possible it could explain this pattern of low heterozygosity if parts of the genome end up in one copy?

Reply: Indeed in the articles cited aneuploidy is pervasive. However, the authors of those studies specifically refer to 'homologous chromosomes from opposite species' so I suspect that the reviewer's suggested mechanism is not responsible for this pattern. We have noted this in the sentence in text.

Reviewer Point R 1.5 — L348: How does ponds and lake environments differ in all those stickleback experiments? While the difference compared to marine environments for example might not be too high, I expect the environment of a small pond might still be quite different from the one of a big lake. In such cases there might be some room for positive selection of some transgressive phenotypes

Reply: We acknowledge that the small ponds do not perfectly capture the ecosystems of large lakes. However, in short-term experiments such as those that we analyze, past results have indicated that they largely provide fish the same diet they eat in the lakes. (Over longer time periods the zooplankton becomes depleted in a way that it doesn't in lakes.) We have added a new sentence reading: "Fish in ponds have diets that are largely representative of what they consume in nature (Arnegard et al. 2014), and there is no evidence supporting the hypothesis that ponds contain novel adaptive peaks." In fact, 'mismatched' fish in Arnegard et al. (2014) did exploit a novel niche of eating mostly springtails, but

it was quite maladaptive. Springtails are terrestrial and are not typically found in abundance in fresh water.

Reviewer 2

Reviewer 2 Major Points

Reviewer Point R 2.1 — In their manuscript titled "Genetic evidence for environment-dependent hybrid incompatibilities in threespine stickleback" Thompson et al. explore the genomic evidence for sets of trait mismatches in hybrids that reduce fitness (what the authors refer to as 'ecological incompatibilities'). This paper presents a creative follow-up to a previously published meta-analysis of trait distributions (and their potential fitness implications) in hybrids (Thompson et al. 2021), and integrates (mostly) previously published genetic/genomic datasets to assess if hybrids reared in semi-wild conditions experience selection against mixed ancestry and subsequent increased heterozygosity more so than hybrids reared in the lab.

Overall I thoroughly enjoyed the manuscript and feel this work provides much needed perspective. I also applaud the authors for a creative use of previously published data. The work makes a novel and, in my opinion, very important contribution to the speciation literature. It is also well written and extremely thorough in analysis. I applaud the authors (again!) for the transparency of the data and methodology. Although my review is overall very positive, I do have some concerns that I think could be addressed with written caveats or minor additional analyses.

Reply: We are glad the reviewer enjoyed reading our manuscript and found that our work represents an important and novel contribution. We also appreciate the thoughtful critique to which we have responded below.

Reviewer Point R 2.2 — 1. The authors make the argument that environment-dependent heterosis is unlikely to explain the small increase in heterozygosity in pond populations versus lab reared populations of stickleback hybrids (lines 278-327), although they do acknowledge that "Ultimately, the data presented here have limited ability to conclusively distinguish between single-locus processes like heterosis and multi-locus processes like incompatibilities" (lines 281-284). While I mainly find their arguments against environment-dependent heterosis compelling, there is one scenario that I think is unaccounted for in the current manuscript, which is environment-specific inbreeding depression (given full sibling mating from wild collected grandparents).

For example, imagine a scenario in which one or both wild-caught parents harbors recessive deleterious alleles in heterozygous form (perhaps at many loci). Given that these alleles are predicted to be relatively common overall, but rare for a given locus, F1s are also likely to be heterozygous for any given recessive deleterious allele. However, if full sibling F1s are mated, then F2s have a high probability of inheriting both recessive deleterious alleles, and expressing some manifestation of inbreeding depression.

In this case, the lines of evidence against environment-specific heterosis are not as compelling: (1) we do not expect that F1s should show heterosis/ increased fitness (recessive deleterious alleles are equally masked in both F1s and in wild parents). (2) Condition might not correlate with overall heterozygosity (just heterozygosity at specific regions of the genome) and/or the inbreeding

depression in question might not affect overall condition. (3) I find the argument that excess heterozygosity should decline with further deviations from a hybrid index of 0.5 (and the data presented) fairly weak and unconvincing overall, particularly as these models are indistinguishable when accounting for the vastly different sample size via bootstrapping. Furthermore, if there is any epistatic variation for environment-specific inbreeding depression, one might expect the same results as under a model of 'ecological incompatibilities'. (4) The data from re-analyzed from Arnegard et al. is quite compelling, although I think this particular argument might benefit from subsampling the 'not A' ground to be the same size as the 'A' group, and asking how often these two classes differ from each other (and from 0).

Reply: We appreciate this thorough comment and agree that environment-specific inbreeding depression is a valid possibility that we should account for. We can use data from Arnegard et al. (2014) to evaluate this possibility empirically. Specifically, the data from Arnegard et al. (2014) have F_2 hybrids whose parents were either (i) siblings ($n = 74$) or (ii) unrelated ($n = 541$). If environment-specific inbreeding were involved we would expect their mean excess heterozygosity to differ and specifically for individuals whose parents are fully siblings to have higher values.

We find that there is in fact no difference in mean excess heterozygosity between the two groups, and the mean (though, again, not significantly different) is actually higher in the 'outbred' group. Thus, we suggest that there is no evidence for environment-specific inbreeding in our data.

These analyses are presented fully in a new Discussion paragraph (starting at line 230) and reference a corresponding figure in the supplement (Fig. S6).

Reviewer Point R 2.3 — [Their point '2.'] This is not an issue *per se*, but it seems the authors are really wanting to detect whether hybrids have a dearth of pairs of alleles with alternate homozygous ancestry, with an increase in heterozygosity being a natural side effect of this. I wonder if a complimentary analysis would be to calculate some modified hybrid index using only loci that are homozygous within an individual and ask if surviving individuals are skewed to one parent or the other at homozygous regions in the pond versus lab. To avoid any issues caused by directional selection, the authors would need to find skew in both directions (i.e. for both parents) within their dataset(s). I think this type of analysis might also quell some worries about the role of inbreeding depression (and other explanations for environment-specific heterosis) in their dataset, if it's possible to do.

Reply: The types of interactions that we are assessing might be pairwise but might be much more complicated, involving dozens of loci. As a result, we are not certain about which conclusions could be drawn from this analysis beyond the heterozygosity–hybrid index relationship has already documented in Fig. S5. Because arriving at sound conclusions and hypotheses might greatly delay our re-submission we have elected to not perform the suggested analysis but we would be happy to take the time to do it if the reviewer felt it was important.

Reviewer Point R 2.4 — [Their point '3'] I think the method presented by the authors is, overall, of great value to the field, and as previously stated, this work has creatively re-used previously collected data to say something very new, and in my mind, of utmost importance. However, while the authors elucidate throughout the manuscript that ability to detect differences in heterozygosity between field vs lab reared hybrids at these genome-wide coarse scales will depend largely on the polygenic nature of the traits of interest and the scale of additivity/dominance for the alleles

controlling those traits. I think the authors should perhaps highlight this caveat under the 'Outlook, caveats, and conclusions' section.

Reply: We have added the following sentence to the final paragraph of the manuscript:

"Moreover, the degree to which mismatched trait combinations are expressed in hybrids, and thus the ability to detect the coarse signal of ecological incompatibilities in an F_2 cross, depends considerably on the underlying genetic architecture of adaptive divergence—in particular the number, effect sizes, and dominance of QTL." (Line 304)

Reviewer 2 Minor Points

I have many, relatively minor and surely picky points that I think would improve the clarity of the manuscript:

Reviewer Point R 2.5 — Lines 43-45: For the severity of 'intrinsic' incompatibilities varying across environments, I also suggest referencing Bundus et al. 2015. Gametic selection, developmental trajectories, and extrinsic heterogeneity in Haldane's Rule. Evolution.

Reply: We've cited this paper where recommended.

Reviewer Point R 2.6 — Line 46: "the number of intrinsic incompatibilities" feels ambiguous to me, because it is unclear whether the authors are referring to the number of interacting loci (as the references would suggest) or the number of unique incompatibilities (which may or may not influence the same hybrid phenotype).

Reply: Thank you for pointing this out, we agree it was ambiguous as written. We've clarified the language here, to say specifically 'the number of loci involved in' incompatibilities.

Reviewer Point R 2.7 — Line 48: I would also reference Chae et al. 2014. Species-wide genetic incompatibility analysis identifies immune genes as hot spots of deleterious epistasis. Cell.

Reply: We have added the reference and appreciate the suggestion.

Reviewer Point R 2.8 — Lines 51-55: I might include the example of extreme transgressive trait combinations and developmental mismatches outlined in Coughlan et al. 2021. The genetic architecture and evolution of life history divergence among perennials in the Mimulus guttatus species complex. Proc B.

Reply: We've included this citation where recommended.

Reviewer Point R 2.9 — Lines 59-60: I find this sentence a bit odd, as the discussion of how polygenic traits are depends, in part, on what type of selection is acting on them. While there is good reason (and evidence) to suggest that Rockman's 2012 piece describes quantitative traits that under stabilizing selection within populations (or even within species), there are also good theoretical reasons to think that the genetic architecture of divergence should be much less complex (see Remington 2015. Alleles versus mutations: Understanding the evolution of genetic architecture requires a molecular perspective on allelic origins. Evolution). Of course, the dearth of

genetic studies quantifying the genetic architecture of species divergence and its relation to fitness make these two world views somewhat difficult to assess, but to date there are many studies which have shown that species divergence can be explained by a fairly small handful of loci. This has implications for how effective this differential heterozygosity test will be (see above).

Reply: We have revised the sentence to make it clear that we are not saying that QTL should be small, but rather that the ability to detect incompatibilities between traits depends on genetic architecture. We've also added the reference to Remington 2015.

Reviewer Point R 2.10 — Lines 210: is this really variation in recombination, or is it variation in independent assortment of chromosomes and/or random chance in which product of meiosis is destined to be the egg (in the case of female meiosis).

Reply: We do mean recombination but absolutely the assortment of chromosomes during meiosis will be very important in addition to recombination—we've added a reference to chromosome segregation to the sentence.

Reviewer Point R 2.11 — Lines 214-216: Could the authors provide a supplemental figure with the raw, observed heterozygosity data plotted? Similarly, could the authors add the data from the three lab studies and Schluter et al. 2021 to Figure S3? I think this would better allow readers to look at the variance among lab studies and marine studies as well. (The authors could even add each as a separate facet/panel so as to avoid confusion by comparing between different crosses and/or environments).

Reply: We've added a new supplementary figure (Fig. S11) showing the observed heterozygosity results. We've elected to show the residuals from our model (very similar to the raw data) in the supper panels because this represents our statistical tests accurately. The raw data are in the lower panel, and in other formats elsewhere in the de Finetti diagrams (although we acknowledge that the number of datapoints can obscure patterns).

We've also made a new panel in Fig. 2 that should allow the readers to really see all the variation within and between studies. This figure shows all populations in the same panel and uses the raw data not data from a model. We have shown excess ancestry heterozygosity here because that is the response variable in our analyses though again the two main alternative response variables (observed ancestry heterozygosity and excess ancestry heterozygosity for loci rather than individuals) are shown in Figs. S11 and S12.

Reviewer Point R 2.12 — Lines 218-230 (Data Analysis section)- do the authors think it would be beneficial to do some sort of bootstrapping of the pond data so that sample sizes are matched between pond and aquaria (which overall have much lower sample sizes) to see how often pond heterozygosity is statistically elevated from 0?

Reply: In our initial submission we presented one-sample t-tests even though our main inference was based on a model comparing the two environments. We meant to convey to the reader that the inferences are the same from the model as from the t-tests. However, this analysis might have given the impression that our failure to reject the null for the lab studies was important for our conclusions, when it was not. Certainly, increasing the sample sizes of these datasets via bootstrapping would make it more likely to detect a difference from 0 but bootstrapping a fairly small sample with a large standard

error on the mean is something else entirely from collecting more data which would narrow the SEM. We have removed the t-tests from our results section and now just focus on the model results (and also have a new panel in Fig. 2 showing all the data for each unique data source) which we hope convinces the reviewer that the bootstrapping is not necessary Of course, we are happy to do it if the reviewer feels strongly.

Reviewer Point R 2.13 — Throughout the manuscript the authors suggest that mismatched homozygous ancestry is resulting in trait mismatches (which in turn have fitness consequences). Although the some of the authors have previously shown the importance of trait mismatches in hybrid fitness (for example, Thompson et al. 2021), I would be cautious to so definitely say that the reduction in fitness is due explicitly to trait mismatches vs transgressive values of single traits (due to epistatic interactions, for example).

Reply: This is a valid point. Rather than say 'mismatch or maladaptive transgression' throughout the manuscript, we have added a couple of sentences to discussion noting that both phenomena have the same genetic basis. It reads: "We also note that selection against mismatched combinations of traits has the same genetic basis as selection against single phenotypes that express maladaptive transgressive values after hybridization (Rieseberg et al., 1999; Barton, 2001; Thompson, 2020). Thus, studies focused on only a single trait under stabilizing selection might still find selection against incompatibilities if hybrids have trait values that are below or above the optimum value (Ono et al., 2017)." (Starting Line 263)

Reviewer Point R 2.14 — I just want to reiterate that the link to phenotype, as described in lines 317-327 is absolutely lovely- huge congrats to the authors for thinking of this.

Reply: Thank you! Author Yaniv Brandvain deserves credit for this one.

Reviewer Point R 2.15 — Lines 339-342: the authors might also find the following reference useful: Smukowski Heil et al. 2019. Temperature preference can bias parental genome retention during hybrid evoluition. PLoS genetics.

Reply: We have cited the paper where recommended.

Reviewer 3

Reviewer Point R 3.1 — The authors present a new analysis of numerous previous pond and lab experiments measuring the survival of stickleback hybrid crosses. They make the fascinating observation that heterozygosity is about 3% higher in pond-raised stickleback than lab-raised stickleback in both benthic x limnetic crosses and marine x freshwater crosses. They conclude that this pattern is consistent with extrinsic genetic incompatibilities, i.e. field selection against incompatible interacting homozygous loci from different parental lines which does not occur in the lab environment. They reject the hypothesis that heterosis in field environments better explains the observed pattern, i.e. hybrids with more heterozygous loci are more fit in ponds.

I think this is a fascinating observation that will inspire numerous field biologists to conduct similar studies or re-examine their old data to look for this pattern. It is also open to numerous

interpretations - the strongest alternative being heterosis/balancing selection. The authors are careful to discuss this alternative explanation that heterozygous loci are directly selected for in field environments and offer several arguments in favor of genetic incompatibilities. Indeed, their conclusion is careful in stating that the observed heterozygosity "is consistent with" extrinsic genetic incompatibilities. I agree that it is consistent, but I think the authors could be more balanced throughout their presentation - particularly in the title, abstract, and introduction. In my view, they do not present explicit evidence for extrinsic genetic incompatibilities - the pattern could also be explained by simple heterosis in field environments.

Reply: We thank the reviewer for their thoughtful comments on our paper and are glad they found our observations novel and fascinating. As the reviewer notes we did try to be careful in how much certainty we projected regarding our findings, and take their point that this tone could be made clearer in other parts of the manuscript.

We have changed the title of the article to "Genetic data suggest that hybrid incompatibilities in threespine stickleback are environment-dependent". We have made many edits to the abstract, introduction, and other parts of the paper (too numerous to list here but are visible in the tracked changes version of the MS) to make clear that our evidence is indirect. Although we do attempt to evaluate all possible alternative arguments for the pattern, we absolutely acknowledge that we cannot be certain about the causal mechanism.

Reviewer Point R 3.2 — The analyses presented are relatively short, although they do explore some simulations. I was also left wondering - how many genetic incompatibilities should we expect in these crosses and are they enough to be predominant in the genome-wide signal of 3% increased heterozygosity? The incredible replication across different crosses, genotyping designs, and studies is impressive and some of these supplemental results should be included in the main manuscript if possible.

Reply: Unfortunately, we cannot begin to speculate about how many incompatibilities there are. We comment in the discussion (when referencing the simulation results) that the answer can vary by many (many) orders of magnitude depending on the assumptions made.

Reviewer Point R 3.3 — I would expect many more incompatibilities in the more divergent marine x freshwater crosses than within-lake benthic x limnetic crosses, particularly given the snowball effect, however excess heterozygosity doesn't appear to increase in more divergent crosses? Why not?

Reply: In our original manuscript, we could have made it clearer that the two crosses are actually fairly similar in how phenotypically divergent they are. In our revision, we highlight that phenotypic divergence in the two crosses might be fairly similar. The new text reads: **The benthic × limnetic crosses are between specialist populations that are at extreme opposite ends of the range of trophic phenotypes observed among this region's native stickleback in fresh water. The marine × freshwater cross involves a limnetic-like marine population and a generalist freshwater population (Cranby Lake) that is intermediate between the limnetic and benthic populations (Schluter 1992; Chhina et al 2021), and is therefore a less divergent cross with respect to trophic characters—however the populations differ in other traits involved in marine-freshwater divergence (Bell and Foster 1994).**

Reviewer Point R 3.4 — I also think the authors could compare different types of heterozygous sites to better test their hypotheses. Is excess heterozygosity still observed in gene desert regions or only genic/near-gene regions? Do gene regulatory regions and/or coding regions exhibit more excess heterozygosity? The authors would have to postulate additional reasons for different site patterns between heterosis and genetic incompatibilities, but this could still point in useful directions for sorting alternative explanations.

Reply: Unfortunately, we do not have data with sufficient resolution to test such hypotheses. We do plan to tackle this exactly in our future work, however (stay tuned)!

Reviewer Point R 3.5 — Finally, all the pond hybrids were the result of natural assortative mating events in the field whereas all the lab hybrids were artificial crosses? As I note below, are the authors worried about the effects of increased inter-F1 family assortative mating resulting in increased heterozygosity in ponds? More detail and controls for lab artificial crossing schemes would be useful.

Reply: The reviewer is correct that all pond hybrids were from natural mating and all labs were crosses. See our response to reviewer two regarding inter-family mating affecting heterozygosity (**Reviewer Point R2.2**)—we hope that response and our new text in the MS addresses this particular concern. We have added some additional new text to the manuscript further highlighting that crosses were between full sibs in the lab but were often 'outbred' in ponds. It reads: "In most cases, F_2 hybrids were produced via mating between full siblings in both the lab and field."

Reviewer Point R 3.6 — Similarly, models exploring the effect of different genetic markers on heterozygosity would also be useful. RADseq has a well known bias against detection of heterozygous sites due to restriction-site dropout (Arnold et al. 2013).

Reply: Following the Editor's comments (**Editor point E 1.1**) we have not taken the suggestion to model different types of genetic markers here. We believe this to be justified because studies using different methods (genotyping array vs. RAD-seq) arrived at the same empirical conclusions. We have, however, taken the latter suggestion in this comment (citing Arnold et al. 2013) and added a couple sentences on the limitations of RAD-seq.

Reviewer 3 Minor Points

Reviewer Point R 3.7 — Line 26: Could the authors qualify this a bit more? I suppose they don't consider tests of postzygotic extrinsic isolation to be tests of hybrid incompatibilities?

Reply: We have added the qualifier 'using sequence data' to the sentence. The reviewer is exactly right—the observation of extrinsic selection against hybrids can be underpinned by different mechanisms from underdominance to incompatibility and sequences are needed to distinguish them.

Reviewer Point R 3.8 — Line 32: Space is limited, but it would be good to know why heterosis is an unlikely explanation? The authors could be much more balanced throughout in presenting heterosis as a plausible additional explanation.

Reply: We have added a sentence stating 'Because heterozygous F_1 hybrids have lower fitness than non-hybrids in the field...' to the abstract. We have also made numerous edits throughout the MS (too numerous to list here but visible on the tracked changes) to be more circumspect about heterosis.

Reviewer Point R 3.9 — Also, I think the authors should clarify in line 30 that this excess heterozygosity could also be a signature of heterosis, not just selection against incompatibilities as they claim, correct?

Reply: We agree that this is an important clarification. Due to space limitations (or perhaps a lack of creativity), we hope that our revised sentence addressing heterosis, which occurs two sentences after the focal line, suffices here.

Reviewer Point R 3.10 — Line 35: incompatibilities or environment-dependent heterosis?

Reply: We believe our original sentence accurately reflects our meaning.

Reviewer Point R 3.11 — Line 76: How does this scale with the genetic architecture of the phenotype? What happens for more polygenic traits if opposite homozygous ancestry is not the predominant pattern?

Reply: We have clarified that this model applies only to cases where the QTL underlying adaptive divergence have 'have non-negligible effect sizes". As traits become more polygenic, the magnitude of trait mismatch is expected to decline. See also our response to **Reviewer Point R2.4**.

Reviewer Point R 3.12 — Line 88: The authors are also assuming some disruptive selection on trait-matched hybrids to get the saddle shape? As stated, this suggests a ridge, not a saddle.

Reply: We have clarified the sentence to "saddle- or ridge-like" because it is the axis orthogonal to the axis of parental divergence on which we want the reader to focus.

Reviewer Point R 3.13 — Line 97: I'm not convinced this is 'compelling' support for extrinsic incompatibilities because there are alternative explanations for the observed pattern of increased heterozygosity in field samples.

Reply: We have removed the word 'compelling' and instead have changed it to 'consistent with'. We meant to use the word 'compelling' in the sense of 'intriguing' but appreciate that it can be read in a more forceful and confident way.

Reviewer Point R 3.14 — Line 126: This looks thorough, but can the authors also run a glm with the effects of experiment type, genotyping method, and their interaction on heterozygosity levels? It would be good to know if the magnitude of increased heterozygosity observed in the field environments differs across different studies?

Reply: We have fit the model as the reviewer suggested. The only term that we can evaluate in this model is genotyping method, and the chi-squared test-statistic is non-significant ($\chi^2 = 0.0434, P = 0.835$). The model is in the 'empirical analysis' code.

Reviewer Point R 3.15 — Line 136: So, all field experiment hybrids were the result of natural assortative mating whereas all lab 'control' hybrids were random crosses with no assortative mating? Since there are multiple F1 families in each experiment, was there increased inter-family assortative mating?

I agree that only considering fixed differences between parents in the F2 generation probably makes this a moot point, but could assortative mating also potentially explain an increase in the survival of more heterozygous fish in fish enclosures? E.g. increased assortative pairings between F1 families in pond experiments could result in more outbred F2 offspring compared to random lab pairings?

Reply: This is an insightful point also noted by reviewer 2. See our response to their point **R2.2**

Reviewer Point R 3.16 — Line 222: An additional model should include the effect of genotyping method (GBS, etc.). Could also include models with effects of sex, presence/absence cutthroat trout, assortative mating/or artificial crosses in the lab to see which model best explains heterozygosity patterns?

Reply: See our response to Reviewer Point *R 3.14*. In our data, treatments (except sex) are not replicated across environments (pond vs. lab) we cannot run the suggested analyses. Because fish are at different ages when sampled (some are juveniles and some are adults) and some studies only have one sex. As a result, we would prefer to not analyse this variable since we do not have any hypotheses about why sex would affect selection on ancestry heterozygosity, and we would like to keep the manuscript as simple as possible and based on a clear hypothesis test. If the reviewer feels strongly, we will be happy to include this analysis, however.

Reviewer Point R 3.17 — Line 295: Is this true - there was zero mortality in the lab before juveniles were released into field enclosures? Heterosis could also act on increased viability of eggs - were there zero egg deaths in lab reared clutches?

Reply: We do not have data on embryo viability for the specific crosses genotyped herein. Most pond-raised fish were born in ponds so this information is not available. Many studies of similar crosses in the lab find well above 95 % viability and no difference between hybrids and non-hybrids. See the Tables in cited studies by Hatfield and McPhail for details.

Reviewer Point R 3.18 — Line 305: remove extra 'which'

Reply: Thank you—we've removed the word.

Reviewer Point R 3.19 — Line 312: This is a very interesting argument in support of the author's extrinsic genetic incompatibilities argument. Should we really expect the fitness effects of genetic incompatibilities to decline linearly from 0.5? Also, why isn't a quadratic fit used as fitness should decline in either direction away from 0.5 ancestry?

Reply: It is unknown whether the fitness declines should be linear or non-linear, but these data are consistent with non-linear effects. We used a linear fit instead of quadratic because the pattern is quite noisy and a simple linear model is simpler than a quadratic model after taking the absolute deviation from 0.5.

Reviewer Point R 3.20 — Line 367: Citations would be useful here, too?

Reply: The beginning of the sentence references the archived R scripts, which is noted in text, rather than insights gleaned from the literature. Our preference is to not dig into the methods of these simulations in the manuscript but we can do so if the reviewer wishes.

Reviewer Point R 3.21 — Fig. 1 could probably be expanded with at least on additional panel. E.g. what are the effects of selection on trait mismatch and heterozygosity? How does heterozygosity relate to fitness effects of genetic incompatibilities vs. heterosis (see my previous comment)?

Reply: With respect to point 1, we believe that panel (b), showing the negative relationship between trait mismatch and heterozygosity, makes the relationship between fitness (negatively correlated with mismatch) and heterozygosity clear. We are unable to estimate the specific fitness effects of mismatch except to say that their rank-order is likely correlated. Thus, we have elected to leave the figure as is. If the reviewer feels strongly, we are happy to make the change.

Reviewer Point R 3.22 — Looking at Fig. 2, I wonder if this pattern is mostly/entirely driven by the tails of the distribution in pond experiments? Would ranked analyses produce similar results to parametric results? Some fish are approaching 40% heterozygous ancestry? What is the predicted vs. observed frequency of these individuals in an F1 intercross (the design of these studies)?

Reply: For both BxL and MxF crosses the differences between pond-raised and lab-raised fish remain significant when a non-parametric (Mann-Whitney U Test) is used. We will leave these analyses in our archived R script but we do not mention them in text in order to minimize the distraction from the main result. With respect to estimating the frequencies of particular genotypes and genotype combinations, we believe this to be beyond the scope of the paper because we would have to consider details regarding recombination and linkage to generate an accurate estimate. We show the distribution of all data most clearly in Fig. S2, and we hope that this figure makes clear that there are no extreme differences in the distribution of datapoints (indivual fish) among treatments or crosses.
